# Uncovering the complexities of biological structures with network-based learning: An application in SARS-CoV-2

**DOI:** 10.1016/j.patter.2021.100259

**Published:** 2021-04-15

**Authors:** Faraz Faghri, Mike A. Nalls

**Affiliations:** 1 Laboratory of Neurogenetics, National Institute on Aging, National Institutes of Health, Bethesda, MD 20892, USA; 2 Center for Alzheimer’s and Related Dementias, National Institute on Aging, National Institutes of Health, Bethesda, MD 20892, USA; 3 Data Tecnica International, Glen Echo, MD 20812, USA

## Abstract

Network-based learning enables the identification of possible undiscovered interactions in biological systems. In this issue of *Patterns*, Du et al. show that applying these methods to severe acute respiratory syndrome coronavirus 2 (SARS-CoV-2) reveals potential infection targets of the virus and possible interactions between SARS-CoV-2 proteins and human proteins.

## Main text

Biological data embody a significant amount of information that is not easily represented in traditional data structures such as tabular data. Network-based data structures and methods have been an effective way of incorporating relational information and describing complex biological systems. In this issue of *Patterns*, Du et al. used network-based methods to discover novel virus-host interactions underlying the coronavirus disease 2019 (COVID-19) pandemic.^1^

Network-based learning is based on the premise that we can use our current experimentally discovered knowledge to predict novel interactions that have not yet been identified. In this case, our current biological knowledge is structured into a network containing aggregated data from multiple mammals and severe acute respiratory syndrome coronavirus 2 (SARS-CoV-2). The network was organized into two layers: an organism and a protein layer. Four types of interactions were defined: protein-protein (between virus and host protein groups), infection (between virus and host group), similarity (between homologs of proteins), and belonging (between organism layer and protein layer). The final virus-host interaction network comprised 246 nodes and 1,766 interactions.

With the biological network constructed, the goal has been to predict possible undiscovered interactions that can be used to develop treatment and vaccine targets. The interaction prediction problem has been formulated as a classification task in machine learning. The Multi-layer Perceptron (MLP) classification method was used to classify non-studied protein interactions into three classes of (1) protein-protein interaction, (2) infections, and (3) no-interaction. In comparison with classic methods that rely on potentially sparse subject-level data that test various interactions individually, this machine-learning approach considers the comprehensive structure of the network and individual interactions.

By integrating multiple host-virus datasets, this work brings forward an interesting capability to predict novel susceptible hosts, a meaningful application in the context of the ongoing COVID-19 pandemic, and potential new emergent variants. Results revealed five potential infection targets of SARS-CoV-2 and 19 possible interactions between SARS-CoV-2 proteins and human proteins, a useful hypothesis for further experimental validation.

This work has incorporated multiple datasets, and although the network-based methods and results are promising, the reader needs to be mindful of their limitations. Results from network-based methods are possibly skewed based on the quality of input data, level of certainty, and missingness percentage. Our biological knowledge is a product of research discoveries that are driven by funding (funding bias). Publication bias can also be a major issue, inherent in many text-mining studies used for networks or as priors for weights. Therefore, any constructed biological network based on publications is inherently biased. If the network has more information about a particular organism, or knowledge quality was not uniform among subnetworks, the results could be biased toward our preconceptions. For instance, in this work, only 17 mammalian species were included due to data availability.^1^

Ultimately, network-based methods in biomedical research are helpful approaches for generating hypotheses; however, like any method work, these results require more in-depth experimental validation and should be treated with caution as a guiding tool for experiment prioritization. To ensure reproducibility and application to the broader scientific community, transparency in network building and analysis is paramount. In this work, to help an independent replication and extension of the results, the analysis code is made available (https://github.com/hangyu98/IMSP) in a structured and easy-to-follow manner.

This work by Du et al. is a constructive proof of concept demonstrating the value of multi-species data integration. The model can be improved by including more high-quality datasets as well as data modalities (e.g., gene set enrichment analysis and sequence motif analysis). The approach can be extended to domains other than infectious diseases by appropriately redefining the network’s nodes and interactions. However, the results depend on the availability and quality of input data. Thorough investigation is necessary to assess the level of bias in the training data, which is consequently introduced into the model.

There are still many open questions and possible research directions in network-based learning: (1) how to effectively and efficiently provide for user interaction with the network(s) so that they could feed their experiential parameters and results into the model and receive interactive feedback; (2) how to incorporate the demographic diversity in the age, gender, and racial/ethnic composition of the population; (3) how to predict rare interactions which we don’t have much training data for; (4) how to enhance the interpretability of the models and to make them more understandable for the biologists; and (5) other than experimental validation, how to evaluate the robustness and generalizability of these models in biological settings across varying domains.
